# Chemical Exposures: Cats as Sentinel Species

**DOI:** 10.1289/ehp.115-a580a

**Published:** 2007-12

**Authors:** Carol Potera

Pet cats may be like canaries in coal mines when it comes to evaluating the health impacts of polybrominated diphenyl ethers (PBDEs), persistent chemicals used in carpet pads, furniture, and electronics. Chronic PBDE exposure may partly explain an epidemic of hyperthyroid disease in older cats, says Janice Dye, a U.S. EPA research biologist. In turn, studying the effects of chronic PBDE exposure in cats could offer clues as to the effects in their human counterparts.

Veterinarians first noticed a dramatic surge in feline hyperthyroidism (FH) in the 1980s, coinciding with the use of PBDEs as flame retardants in consumer products. FH, the most common endocrine disorder in cats, causes rapid weight loss due to increased concentrations of thyroxine. Histologic changes in FH mirror those seen in older humans experiencing toxic nodular goiter (TNG), in which an enlarged thyroid gland overproduces thyroxine. The causes of FH and TNG remain unknown.

Dye looked at whether hyperthyroidal cats had greater body burdens of PBDEs. She and her colleagues measured numerous PBDE congeners in serum samples collected from 23 cats, 11 of which were positive for FH. They report in the 15 September 2007 *Environmental Science & Technology* that total average PBDE levels were three times higher in older cats with FH than in younger cats without FH, but the difference was not statistically significant because of high within-group variability. “All cats are high [compared with humans], and some cats are incredibly high,” Dye notes. She also found that cat food, particularly canned fish-flavored brands, contains high levels of PBDEs. Cats swallow PBDEs in food and by licking PBDE-laden house dust from their fur.

PBDE congeners contain bromine atoms that mimic the iodine in thyroxine. PBDEs may interact with thyroid-binding proteins to displace thyroxine, and chronic exposure may lead to sustained increases in secretion of thyroid-stimulating hormone and thyroid hyperplasia. “When that happens,” says Dye, “it’s pedal to the metal, and the cat’s thyroid makes too much thyroxine.”

The potential link between FH and PBDEs suggests that house cats may be sentinels for chronic indoor PBDE exposure in people. By understanding more completely how PBDEs alter thyroid hormone levels in cat sentinels, “we can assess whether comparable risk exists for exposed people, especially children,” Dye says. Like cats, toddlers may be inordinately exposed to PBDEs in dust by crawling on floors and placing objects in their mouths. A case study of one family, described in the October 2006 *EHP*, found that a toddler had PBDE levels up to 10 times higher than those of his parents. “We don’t know what PBDEs do to children during critical stages of development,” says Dye, but cats offer hints about “where chronic exposure may lead.” People in the United States have the highest PBDE levels reported worldwide, according to a meta-analysis published in the 15 February 2004 *Environmental Science & Technology*.

Heather Stapleton, an environmental chemist at Duke University, points out that the link between PBDEs and health problems in cats is reminiscent of the revelation 25 years ago that children ingesting lead in household dust experienced learning problems. Says Stapleton, “It’s important to understand the effects of exposure to these brominated flame retardants in humans.”

## Figures and Tables

**Figure:**
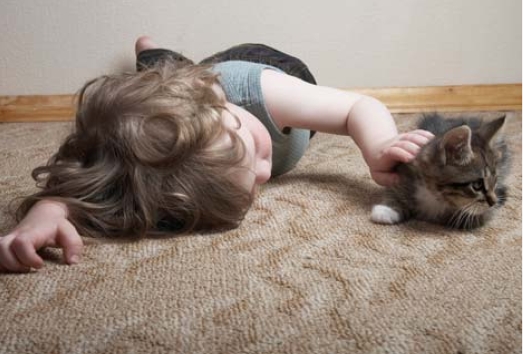
Taking a cue from kitty Health effects seen in cats exposed to PBDEs may offer insights into how these chemicals affect humans.

